# Comparison of dynamic flow interaction methods between pipe system and overland in urban flood analysis

**DOI:** 10.1038/s41598-021-88246-z

**Published:** 2021-06-08

**Authors:** Xiaoli Hao, Yanmin Li, Shu Liu

**Affiliations:** 1 Research Center on Flood & Drought Disaster Reduction of the Ministry of Water Resources, China Institute of Water Resources and Hydropower Research, Yuyuantan South Road, Haidian District, Beijing, China; 2 State Nuclear Electric Power Planning Design & Research Institute, Dijin Road, Haidian District, Beijing, China

**Keywords:** Natural hazards, Hydrology

## Abstract

Urban flooding can be predicted by using different modeling approaches. This study considered different methods of modeling the dynamic flow interactions between pipe systems and surface flooding in urban areas. These approaches can be divided into two categories based on surface runoff collection units. This paper introduces a new hydrodynamic model that couples the storm water management model and the 2D overland model. The model’s efficiency was validated based on the aforementioned experimental dataset; agreement was verified by correlation values above 0.6. Additionally, this study used different approaches and compared their accuracy in predicting flooding patterns. The results show that the use of sub-catchments to model the collection of surface runoff was not predictive of the inundation process, indicating a lower goodness of fit with the recorded values than that of adopting cells. Moreover, to determine which method of adopting cells to collect runoff could better predict rainstorm-induced inundation, an error and correlation analysis was conducted. The analysis found low error and high correlation, suggesting that inundation can be effectively predicted by the new approaches. Ultimately, this study contributes to existing work on numerical analysis of the interaction methods of urban flooding.

## Introduction

The expansion of impermeable land surface in urban areas is positively correlated with the acceleration of urbanization. The development mode for urban drainage networks, characterized by prioritizing “surface construction first and underground construction second,” significantly increases flood hazards in urban areas^1–3^. Numerical models are a crucial technical solution for timely and accurate prediction of flooding. There are many types of two-dimensional (2D) overland surface models which can simulate detailed overland flow processes by utilizing different governing equations, computing cells, and parallelization techniques^4–8^. Their prediction accuracy mostly depends on the resolution of the terrain data^9,10^. Many one-dimensional (1D) models also exist to simulate dynamic flow in urban sewer networks^11^. The 1D/2D interaction flows are complex and transient^12^. The specific interaction method plays a crucial role in the numerical calculations of urban flooding, which explains researchers’ interest in different interaction methods^13–15^.

The two main numerical interaction methods were developed from urban flood models by simplifying the complex physical phenomena involved^16–18^. In one interaction method, sub-catchments are applied to collect the surface runoff into the pipe system^8,19^. The overloaded manholes trigger the 2D overland hydrodynamic model, which simulates the overland inundation process induced by surcharge water from the pipe system. The other adopts overland cells to collect the surface runoff^6,10^ and the dynamics are calculated based on the hydrodynamic method. The exchange nodes collect surface runoff into the pipe system, and in the overloaded state, the overflowing water from the pipeline is directed overland.

Although flow interactions between the two models are of interest to researchers, few studies have focused on comparing the different methods^20–24^. In most research, the linkage between the two models is simplified to consider only the manholes, where it is assumed that the surcharging process occurs immediately. In reality, the inlets collect urban runoff and discharge it into the pipe system under drainage conditions^25^, and the weir and orifice equations are adopted to compute the exchange discharge. Under overloaded conditions, the manhole cover can postpone the overflow process^26^.

In this paper, a new urban flood model is proposed. It combines the 1D pipe system (SWMM) and the 2D overland model (FullSWOF-2D), thus reflecting the complex properties of dynamic exchange flow between the surface and pipe systems. The two models were executed separately, and appropriate linkages were made to convey information gained at specific positions and times. It was assumed that the interaction locations were cells that were suitable for nodes, including inlets and manholes. The numerical modeling results obtained using previous linkage methods were compared, and new approaches that could more accurately simulate the dynamic exchange flow were proposed. Numerical modeling results obtained by different linkage methods, including SWMM/FullSWOF (SF) and FullSWOF/SWMM (FS) methods, were compared. The methodology is described in detail in the next section, after which the model is verified by a full-scale physical experiment. Further, results obtained from different linkage methods are analyzed, including the new approaches introduced in this paper. Finally, we conclude with the main research results and provide recommendations to help professionals choose an appropriate method for urban flood simulation.

## Methodology

### Hydraulic models

#### 1D sewer model

In this paper, the Storm Water Management Model (SWMM 5) used by the United States Environmental Protection Agency (USEPA) was applied to simulate dynamic flow in the pipe system^27^. SWMM is a pipe drainage model, expressed as the combined continuity and momentum equation in the links (Eq. 2) and the continuity equation at the nodes (Eq. 1).

1 $$\frac{\partial H}{\partial x}=\frac{\sum {Q}_{i}}{\omega }$$

2 $$gA\frac{\partial H}{\partial x}-2v\frac{\partial A}{\partial t}-{v}^{2}\frac{\partial A}{\partial x}+\frac{\partial Q}{\partial t}+gA{S}_{f}=0$$

here $H$ is node depth (m), ${Q}_{i}$ is node inflow or outflow discharge (m^3^/s), $\omega$ is free surface area at the node (m^2^), $A$ is pipeline cross-sectional area (m^2^), $v$ is pipeline velocity (m/s), ${S}_{f}$ is friction resistance given by ${S}_{f}={n}^{2}/A{R}^{4/3}Q\left|v\right|$, and *R* is the hydraulic radius (m).

#### 2D Overland flow model

2D flood propagation processes are simulated using the open-source Full Shallow Water equations for Overland Flow (FullSWOF-2D) software developed by the Denis Poisson Institute^28^. The model makes use of the finite volume explicit discretization scheme (Eq. 3), which is superior to the finite difference method because it guarantees both mass conservation and positive water depths.

3 $$\begin{aligned} & \partial_{t} h + \partial_{x} \left( {hu} \right) + \partial_{y} \left( {hv} \right) = Rt - I + Q_{in} \\ & \partial_{t} \left( {hu} \right) + \partial_{x} \left( {hu^{2} + \frac{{gh^{2} }}{2}} \right) + \partial_{y} \left( {huv} \right) = gh\left( {S_{{0_{x} }} - S_{{f_{x} }} } \right) \\ & \partial_{t} \left( {hv} \right) + \partial_{x} \left( {huv} \right) + \partial_{y} \left( {hv^{2} + \frac{{gh^{2} }}{2}} \right) = gh\left( {S_{{0_{y} }} - S_{{f_{y} }} } \right) \\ \end{aligned}$$

here *h* is the surface water depth (m), and $u$ and $v$ are the flow velocities in directions x and y (m/s), respectively. Since the model does not consider erosion, ${S}_{{0}_{x}}$ and ${S}_{{0}_{y}}$ are functions of space ${z}_{b}$, given by ${S}_{{0}_{x}}=-{\partial }_{x}{z}_{b}(x,y)$ and ${S}_{{0}_{y}}=-{\partial }_{y}{z}_{b}(x,y)$, respectively; ${S}_{{f}_{x}}$ and ${S}_{{f}_{y}}$ indicate bed friction, obtained from Manning’s formula ${S}_{{f}_{x}}={n}^{2}u\sqrt{{u}^{2}+{v}^{2}}/{h}^{4/3}$ ,${S}_{{f}_{y}}={n}^{2}v\sqrt{{u}^{2}+{v}^{2}}/{h}^{4/3}$ respectively; $n$ is Manning’s roughness (s m^−1/3^). In Eq. (5), $Rt$ is the rain intensity (m/s), $I$ is the infiltration rate (m/s) determined by the bi-layer Green–Ampt model, and ${Q}_{in}$ is the surcharge discharge linking the sewer model and 2D overflow surface model. Several boundary conditions are available, including the wall, Neumann, periodic, imposed depth, and imposed flow boundary conditions.

### 1D/2D linkage approaches

The interactions between sewer and overland flow include SF methods that apply rainfall to sub-catchment units, and FS methods that apply rainfall to cell units. SF methods are classified into two categories^29^: one-directional flow interaction method (SFO) and bidirectional flow interaction method (SFB). The previous FS method mostly regarded nodes as uniform type (FSU). Based on the consideration of real physical phenomena, this research proposes two new approaches for simulating dynamic exchange flow. In one method, nodes are simplified as the manhole and inlet type (FSMI), where surface runoff flows into sewer networks through inlets, and overflow from sewer networks occurs in the manhole in the overload state. The second one, FSMIC, was developed based on the FSMI method. It considers manhole covers’ function in delaying the overflow process. The simulation methods are described below in detail.

**Figure 1:**
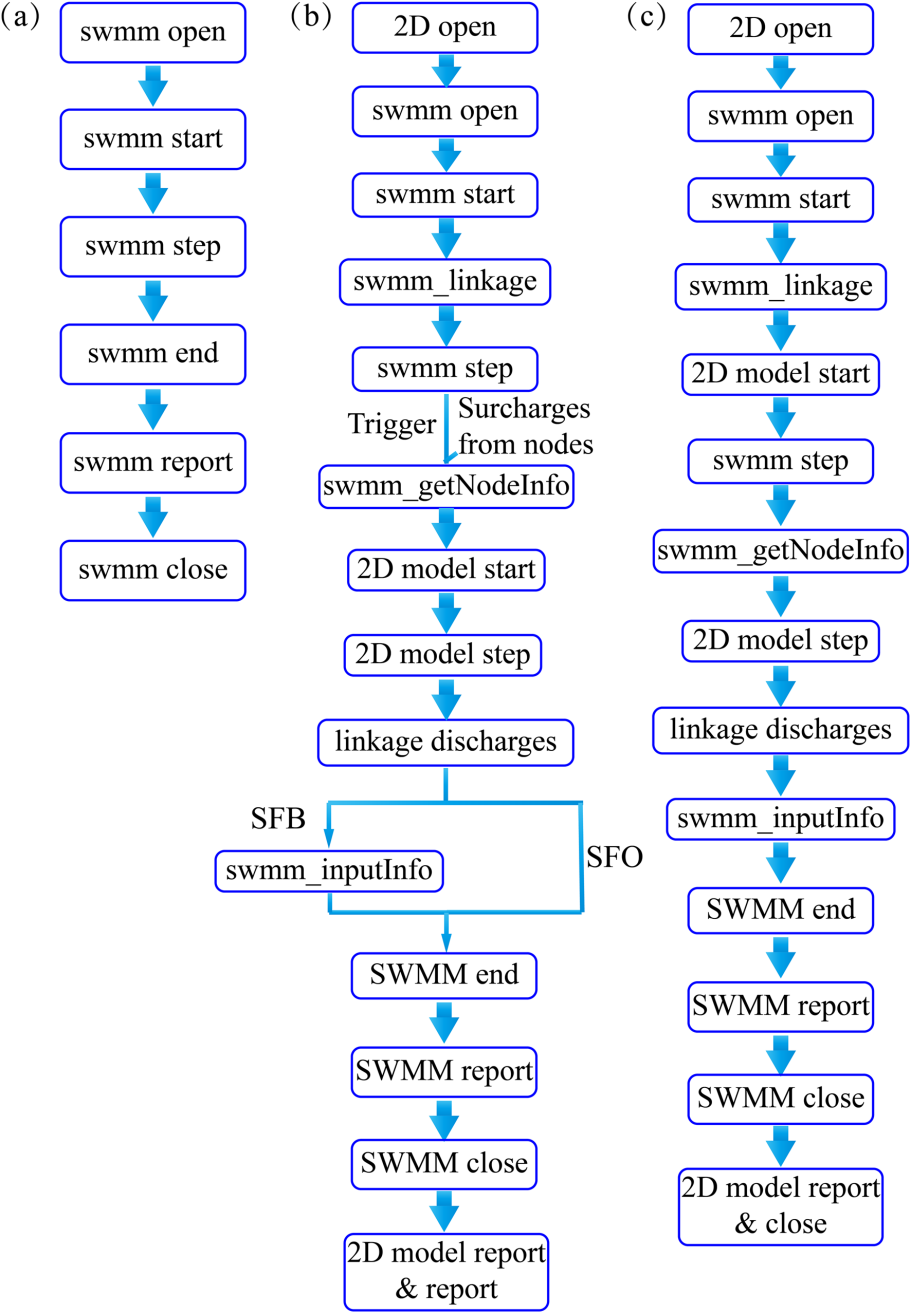
Methodology model structure. (**a**) SWMM original structure. (**b**) SF method structure. (**c**) FS method structure.

The original SWMM5.1 model is categorized into six functions as shown in Fig. 1a, which enables an addition to other models solely for particular usage^30^. To exchange data between the sewer and overland models, this study adopted the linking methodology, including four extra functions: swmm_linkage, swmm_getNodeInfo, swmm_inputInfo, and linkage discharge. The developed swmm_linkage function enables to extract the node detail from SWMM. The swmm_getNodeInfo function conveys the node water depths to the 2D model during every simulation time. The swmm_inputInfo function exchanges the discharge between both models, which was calculated by linkage discharge function in the 2D model. The exchange discharges can be either positive or negative, depending on whether water is being transferred from or to the 2D model. The SF and FS method model structures are displayed as Fig. 1b,c, respectively.

#### SF method

The SF method applies sub-catchments to collect rainfall. The surface runoff is directed into the sewer network by nodes via the runoff module, and the surface runoff is approximated as one-dimensional flow based on the nonlinear reservoir hydrologic formula. However, the surface runoff collection process does not consider the node capacity. The over-capacity discharge from nodes, as obtained from the SWMM simulation, are regarded as trigger points in the 2D model. The pipe system water overflow triggers the 2D model simulation, and over-capacity discharges are calculated using orifice equation. The SFO method merely allows one-directional flow from the pipe system to the surface: return flow from the surface to the pipe system is not permitted, and the SWMM and FullSWOF-2D models are executed sequentially. In comparison, the SFB method allows for bidirectional exchange flow between the two models. The water overflow can return to the pipe system when it arrives at a node that is linked to a pipeline with high capacity. The interaction discharges are calculated the same way as in the FSU method.

#### FS method

The FS method applies cell units to collect rainfall. After the runoff process, the overland flow is directed into the sewer network by exchange nodes. The overflowing water flows from the pipe system to the surface when the discharge exceeds its capacity, but it can also return to the system, which has enough capacity to collect the surface runoff. The FSU method simplifies all exchange nodes as manholes, meaning that all are considered able to collect the surface runoff into the pipe system. In a real-life urban environment, the exchange nodes are classified into inlets and manholes. In the FSMI method, the inlets collect the surface runoff into the sewer network based on rectangular weir and orifice discharge equations, and the overloaded state occurs immediately when the hydraulic head in the manhole reaches the surface water level. The FSMIC method considers the role of manhole covers in delaying the overflow process. In this case, the overload state occurs only after the hydraulic head exceeds the critical pressure head in the manhole.

The bidirectional interaction discharge is calculated based on the water level difference between the two models. During the simulation, the interaction discharges in three different cases are calculated according to the surface water depth ${h}_{2D}$, the hydraulic head at the inlet ${h}_{1D}$, and the ground elevation ${z}_{2D}$.

**Figure 2:**
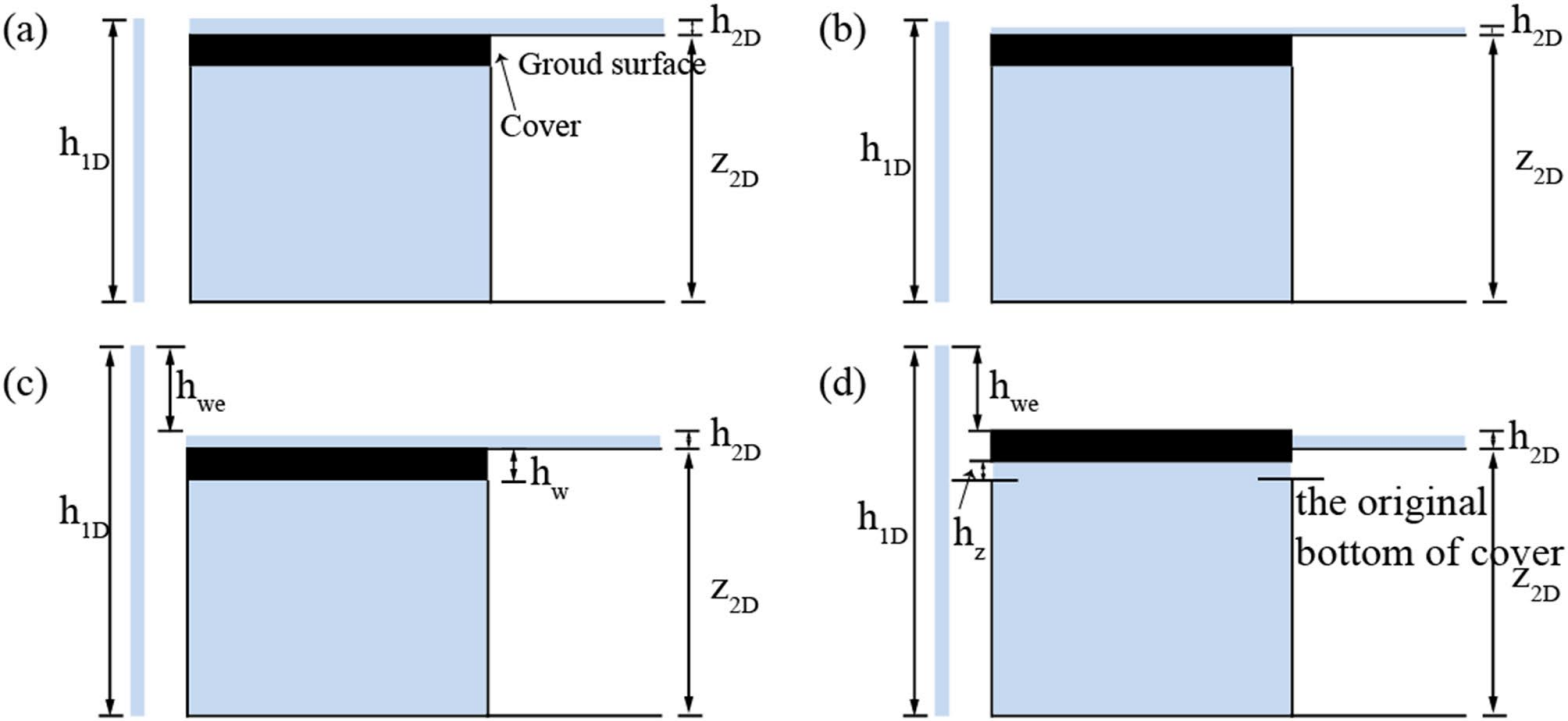
The lifting process of a manhole. (**a**) When ${{h}_{1D}=h}_{2D}+{z}_{2D}$, the overflow process occurs immediately when the head exceeds this critical point in the FSMI method. (**b**) When ${{h}_{1D}>h}_{2D}+{z}_{2D}$, the overflow process does not occur in the FSMIC method. (**c**) The critical pressure head is exactly high enough to lift the manhole cover in the FSMIC method. (**d**) The overflow process occurs in the FSMIC method when the manhole cover is lifted in vertical displacement by the difference between the pressure head and the critical pressure head.

- Drainage condition
  The exchange node discharge is given by the weir Eq. (4) under the drainage condition, or by the orifice Eq. (5) when ${h}_{1D}<{z}_{2D}$ or ${z}_{2D}<{h}_{1D}<{h}_{2D}+{z}_{2D}$.
  4 $$Q={\mathrm{c}}_{\omega }P{h}_{2D}\sqrt{2g{h}_{2D}}$$
  5 $$Q = {\text{c}}_{0} A_{n} \sqrt {2g(h_{2D} + z_{2D} - h_{1D} )}$$
  here $Q$ is the exchange discharge (m^3^/s), whose positive and negative represent the flow from the surface to the pipe and the flow from the pipe to the surface, respectively; ${c}_{\omega }$ represents the weir discharge coefficient; $P$ represents the wetted perimeter;${c}_{0}$ represents the orifice discharge coefficient; and ${A}_{n}$ is the net inlet cross-section area. In the FSU method, ${A}_{n}=\pi {r}^{2}$, $P=2\pi r$, and $r$ are the radius of the exchange node. In the FSMI and FSMIC methods, inlets can collect surface runoff, where $P= 2*(L+B)$, ${ A}_{n}=L*B$ ($L$ is the inlet length and $B$ is the inlet width).
- Surcharge condition
  In the FSU and FSMI methods, the interaction discharges depend on Eq. (6). If ${h}_{1D}={h}_{2D}+{z}_{2D}$, the overflow process occurs immediately when the pressure head exceeds this critical point, as shown in Fig. 2a.
  6 $$Q = - {\text{c}}_{0} A_{n} \sqrt {2g(h_{1D} - h_{2D} - z_{2D} )}$$

In the FSMIC method, if ${h}_{1D}>{h}_{2D}+{z}_{2D}$, the overflow process does not occur if the pressure head is not high enough to lift the manhole cover, as shown in Fig. 2b. Figure 2c shows the critical pressure head ${h}_{1Dc}$ that is exactly high enough to lift the manhole cover, and the overflow process occurs immediately when the pressure head exceeds this critical point. During the overflow process, the manhole cover is lifted in vertical displacement by the difference between the pressure head and the critical head. Overflow discharge is calculated by Eq. (7), as shown in Fig. 2d.

7 $$Q = - {\text{c}}_{0} B_{w} \left( {h_{z} - h_{w} } \right)\sqrt {2g(h_{1D} - z_{2D} - h_{2D} )}$$

here ${B}_{w}$ is the edge perimeter of the manhole cover; the lift-up vertical displacement ${h}_{z}$, given by ${h}_{z}={h}_{1D}-{h}_{1Dc}$, which is the difference between the pressure head ${h}_{1D}$ and the critical pressure head ${h}_{1Dc}$; ${h}_{1Dc}={h}_{2D}+{h}_{we}+{z}_{2D}-{h}_{w}$, ${h}_{w}$ and ${h}_{we}$ are the thickness and equivalent of the manhole cover, respectively; ${h}_{we}$ given by ${W}_{w}/\rho g{A}_{w}$; ${W}_{w}$ is the weight of manhole cover, $\rho$ is the water density, and ${A}_{w}$ is the area of the manhole cover.

### Model rationality analysis method

In this research, the computed and measured values were evaluated by the following methods:

The correlation coefficient, R^2^, measures how well the computed data match the actual data and was defined as a normalized measure to depict the linear correlation between the computed and measured values^31^. The Nash–Sutcliffe efficiency coefficient (NSE) evaluates the relative consistency between the computed and measured data^32,33^. The NSE is an index used to evaluate model precision.

8 $${R}^{2}={\left\{\frac{\sum_{i=1}^{T}\left[{(X}_{m}^{t}-\overline{{X}_{m}}){(X}_{o}^{t}-\overline{{X}_{o}})\right]}{\sqrt{\sum_{i=1}^{T}{{(X}_{m}^{t}-\overline{{X}_{m}})}^{2}}\sqrt{\sum_{i=1}^{T}{{(X}_{o}^{t}-\overline{{X}_{o}})}^{2}}}\right\}}^{2}$$

9 $$NSE=1-\frac{\sum_{i=1}^{T}{{(X}_{o}^{t}-{X}_{m}^{t})}^{2}}{\sum_{i=1}^{T}{{(X}_{o}^{t}-\overline{{X}_{o}})}^{2}}$$

where ${X}_{o}^{t}$ is the measured value at time *t*, ${X}_{m}^{t}$ is the computed value at time *t*, $\overline{{X}_{o}}$ is the average of the measured value, and $\overline{{X}_{m}}$ is the average of the computed value. If R^2^ = 1.0, the computed values fit the measured values perfectly, whereas if R^2^ = 0.0, the computed values fail to fit the actual data. The NSE value is between − ∞ and 1. A high NSE value indicates a better simulation result, while NSE values less than 0 indicate a poor simulation accuracy.

Moreover, the root mean square error (RMSE) and relative peak error (RPE) are adopted to estimate the difference between computed and measured values.

10 $$RMSE=\sqrt{\frac{{\sum }_{i=1}^{n}{\left({h}_{r}^{t}-{h}_{c}^{t}\right)}^{2}}{n}}$$

11 $$RPE=\frac{\left|{h}_{r}^{p}-{h}_{c}^{p}\right|}{{h}_{r}^{p}}\times 100\%$$

here ${h}_{r}^{t}$ and ${h}_{c}^{t}$ are the recorded and computed water depth at *t* time, respectively; $n$ is the number of recorded point; and ${h}_{r}^{p}$ and ${h}_{c}^{p}$ are the recorded and computed maximum water depth, respectively.

The goodness of fit between observed and modeled flood extent was calculated using Eq. (12).

12 $$Fit=\frac{{A}_{O}\cap {A}_{S}}{{A}_{O}\cup {A}_{S}}$$

Fit is suitable for evaluating the validity of inundation models. The value will equal 1 when the observed and simulated values are correlated, and 0 when no intersection area exists. ${A}_{O}$ and ${A}_{S}$ represent the modeled and observed inundation areas, respectively^34^.

## Case studies

The urban model was tested in an experiment consisting of five rainfall and rainfall-runoff events. The testing was conducted in a laboratory environment by Fraga et al. in Spain^35^. Further, in a case study of *Lianhua* Bridge, previous models and new approaches were comparatively analyzed.

### Experimental case study

#### Experimental brief description

**Figure 3:**
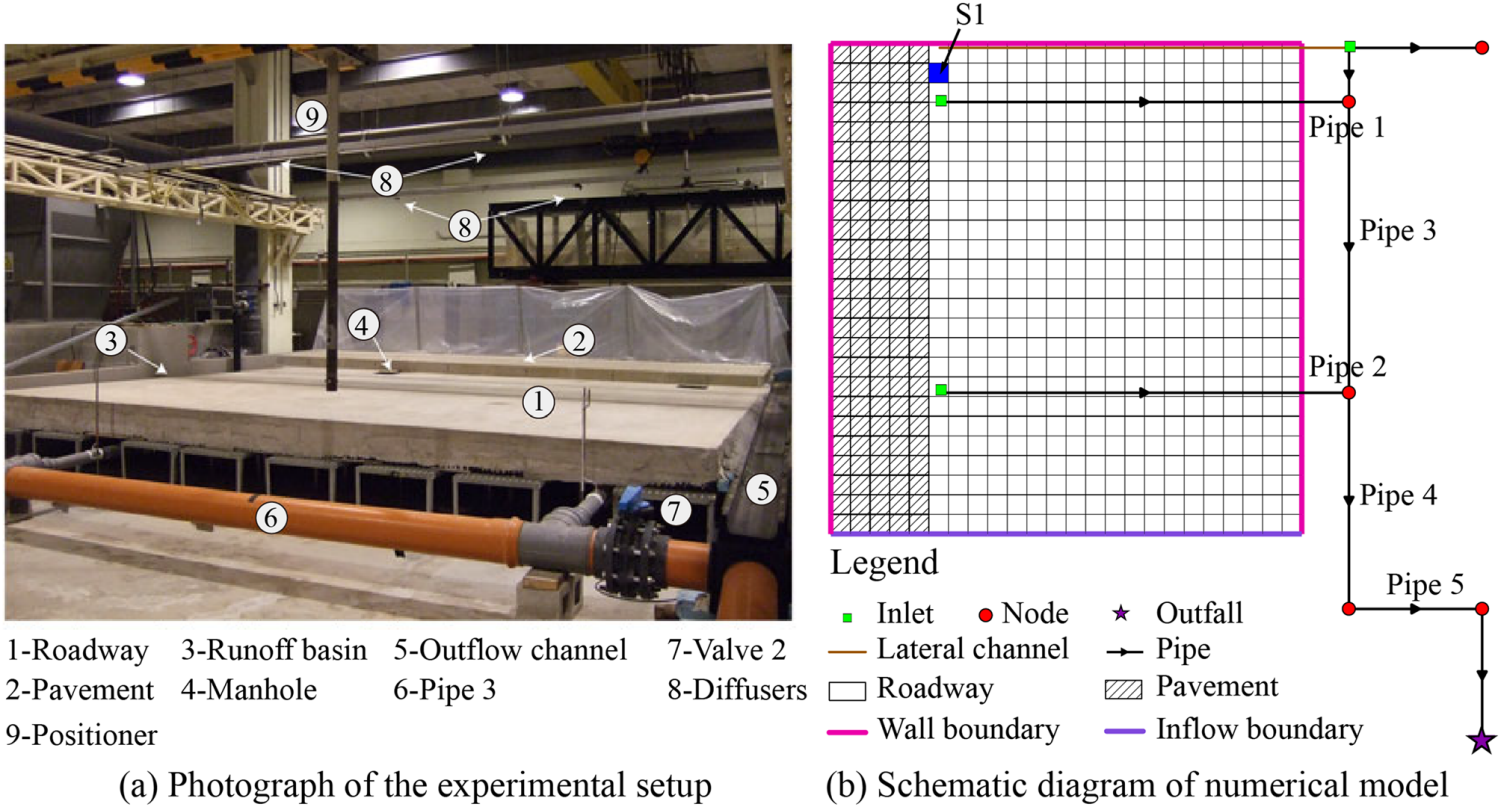
Model construction and configuration for analyzing the drainage model (**a**) Experimental flume^35^. (http://www.tandfonline.com/loi/nurw20). The main components are shown in the picture. (**b**) Numerical model. The arrows represent the slope direction.

To investigate the properties of the urban flood model, a full-scale experimental facility located in the University of A Coruna was used. Figure 3a shows the configuration of the experimental facility, consisting of a section of pavement and concrete roadway. Five experiments were performed to analyze the properties of the model, including three rainfall events, and two rainfall-runoff events. The data are presented in Table 1, where R and RRO stand for rain event and rainfall-runoff event, respectively. Besides, the rainfall events approximately represent rainfall return period based on the designed rainfall process in Beijing, where flood control standard is about 20a. In the rainfall events, rain falls into the concrete surface and the generated direct runoff flow along the terrain. Subsequently, the inlet collects surface runoff and discharges it to the pipe system. In the rainfall-runoff events, a constant discharge was generated by runoff basin as shown in Fig. 3a.

**Table 1:** Five rain events.

Rain event	Mean rainfall intensity (mm/h)	Runoff discharge (l/s)	Rainfall return period
R1	50	0	5a
R2	75	0	20a
R3	90	0	20-50a
RRO1	50	1.4	–
RRO2	75	2.6	–

#### Numerical simulation details

**Figure 4:**
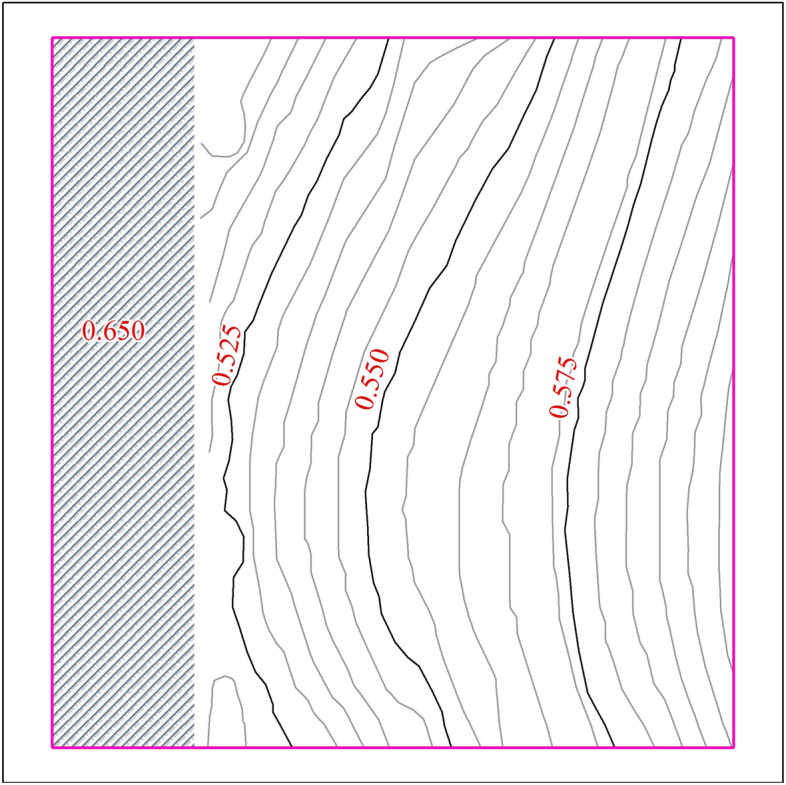
Terrain contour in study area.

In this section, we adopted the FS method in our model to compute the rainfall-runoff event in our study area based on the experimental environment. For the 2D model, the case study considered an terrain contour as shown in Fig. 4. Topographically, in the study area, the part near the inlets was low, whereas the part away from them was high. The size of the study area was approximately 6 m long and 6.25 m wide, which used a uniform regular grid system with cells sized 0.25 × 0.25 m. In the rainfall events, the initial region is supposed to be dry with wall boundary conditions. In the rainfall-runoff events, a constant discharge was provided as an inflow boundary, which was used to generate surface runoff, and is presented by the violet line in Fig. 3b. In this case, the other boundaries were still set as the wall. As shown in Fig. 3b, the pipe system included seven circular pipelines of two different diameters, a rectangular outflow channel, and three inlets. One inlet was positioned at the end of the channel, and two additional inlets collected the surface runoff. The Manning’s roughness value for the pipe system and surface are considered as 0.008 and 0.012 s m^−1/3^ based on the plastic and the concrete material, respectively^36^. This simulation setup involved configuring the solver with a Courant–Friederich–Levy (CFL) value of 0.8. Additionally, a fixed time step (approximately 0.01 s) was obtained from the CFL stability condition in all simulations.

#### Comparison between modeled and measured values

**Figure 5:**
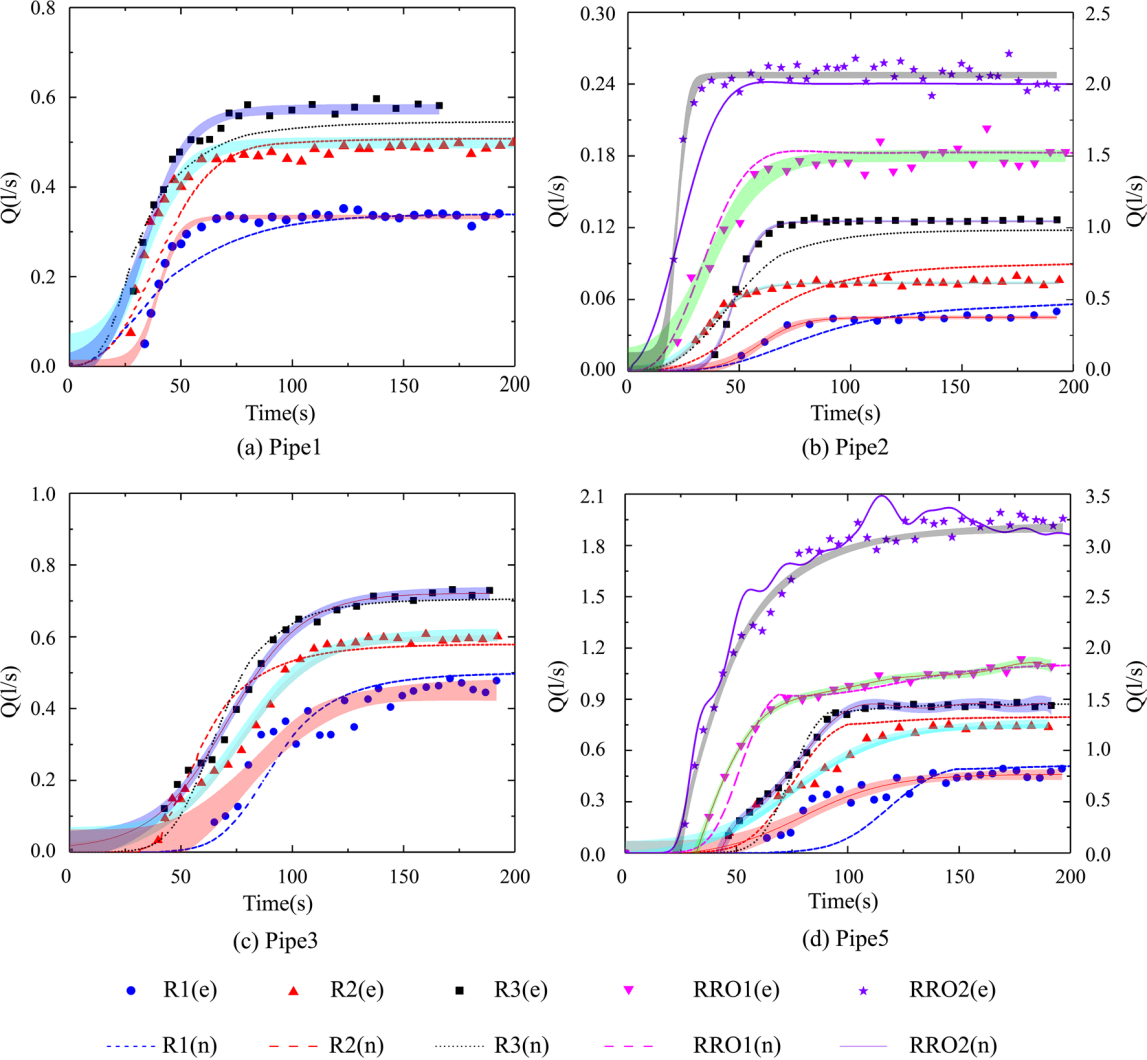
Modeled and measured discharges in the pipe system. The points and lines represent the measured values (e) and the numerical simulation values (n), respectively. There were three rainfall events (R1, R2, R3) and two rainfall-runoff events (RRO1, RRO2). In Fig. b and d, the secondary axis illustrates the discharges in the rainfall-runoff events.

**Figure 6:**
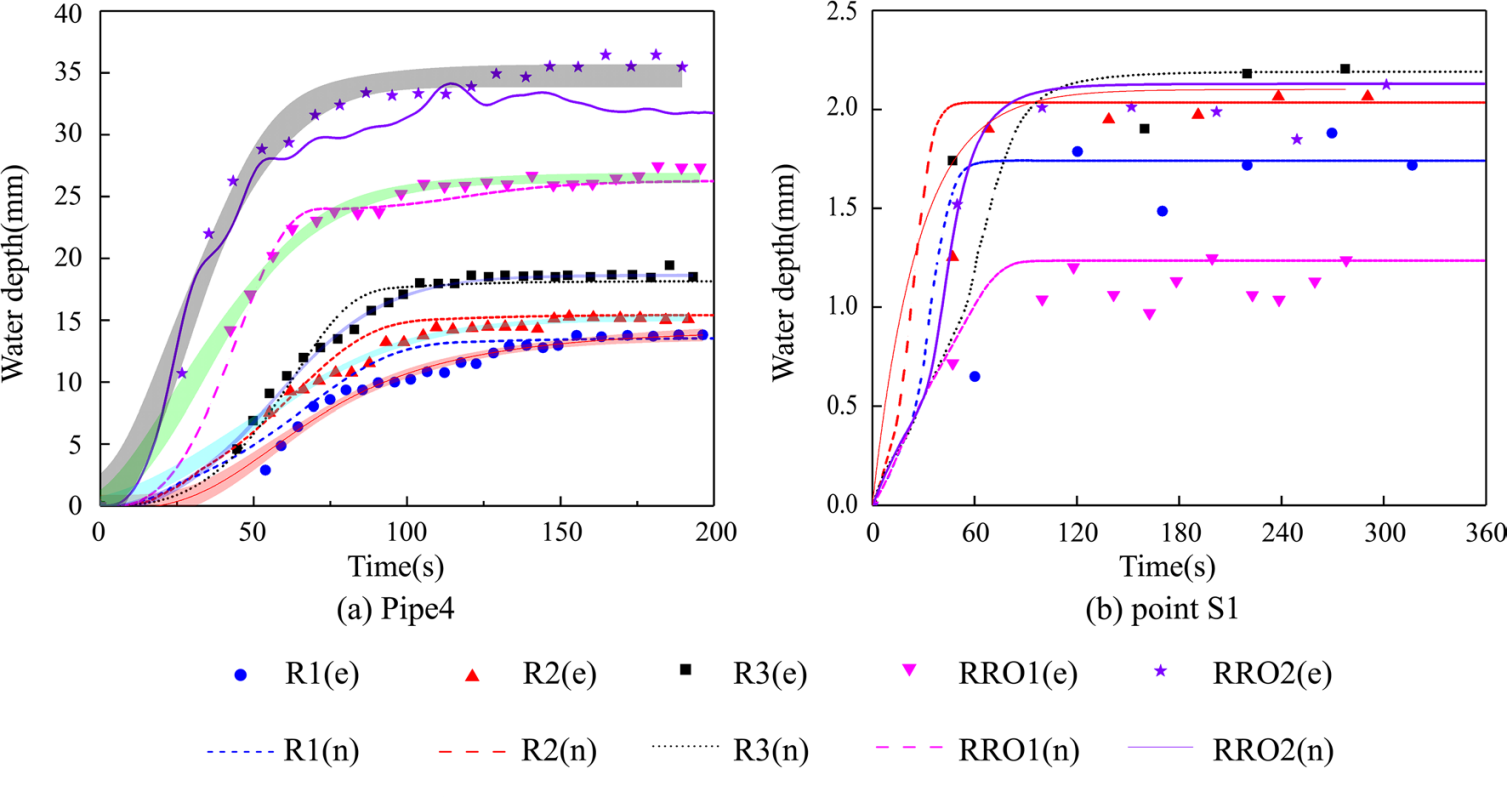
Modeled and measured water depths. (**a**) Pipe 4 values. (**b**) Values for point S1.

A comparison of the modeled and measured values are given in Figs. 5 and 6 under a free-water surface state. In Figs. 5 and 6, the points and lines represent the measured and modeled values, respectively. Moreover, the shadowed areas depict the 95% confidence interval of the measured values, which clarifies the relationship between the computed and measured values. At the beginning of the modeled rainfall events, the numerical results were slightly higher than the measured values in Pipe 1, as illustrated in Fig. 5a. However, as shown in Fig. 5b, the modeled discharges were marginally lower than the measured values in Pipe 2. The modeled and measured values for Pipes 1 and 2 overlapped under the stable condition. Additionally, the modeled discharges were consistent with those measured in the rainfall-runoff events. Figure 5c illustrates ascending discharge values, which differ from the values for other pipes. Meanwhile, the modeled and measured values correlated during the rainfall events. Initially, the modeled discharges were lower than the measured values in Pipe 5; this was due to the differences in the modeled and measured values at the outfall of the pipe system. In other words, differences existed between the basin’s outflow volume and the free outflow volume of the model. Figure 5d illustrates that a good correlation exists between the modeled and measured values for the rainfall-runoff events in Pipe 5 under the stable condition.

Figure 6a depicts that the modeled water depths were initially higher than the measured values in Pipe 4. A correlation between the values is reached in the stable condition. Figure 6b represents the water depth at the located surface point, evolving over time. In the rainfall process, the modeled values were slightly higher than the measured values, and the values correlated in the stable condition. In the rainfall-runoff events, the surface point values were correctly predicted.

To further analyze the simulated results, the results of the correlation analysis were collected in Table 2, which includes the R^2^ and NSE values, as mentioned in “Model rationality analysis method” section. As can be seen from Table 2, all correlation coefficients (R^2^) between the computed and measured values were greater than 0.75 and were in good agreement. All the NSE values were greater than 0.6, which indicates overall agreement between the computed and measured values.

**Table 2:** Results of the correlation analysis.

	R1		R2		R3		RRO1		RRO2	
	R^2^	NSE	R^2^	NSE	R^2^	NSE	R^2^	NSE	R^2^	NSE
Pipe 1	0.798	0.719	0.893	0.756	0.969	0.831	–		–	
Pipe 2	0.819	0.648	0.810	0.653	0.884	0.662	0.910	0.891	0.758	0.634
Pipe 3	0.906	0.738	0.921	0.885	0.957	0.947	–		–	
Pipe 5	0.769	0.647	0.910	0.677	0.980	0.892	0.979	0.931	0.954	0.942
Pipe 4	0.913	0.769	0.917	0.800	0.963	0.950	0.955	0.922	0.918	0.784

In conclusion, the urban model’s efficiency was validated based on the aforementioned experimental dataset. The measured and modeled water depth and discharge values in the pipe system demonstrated a positive correlation, and the surface water depth values were correctly predicted. Ultimately, our urban model proved effective and accurate.

### Real-life case study

#### Study area

**Figure 7:**
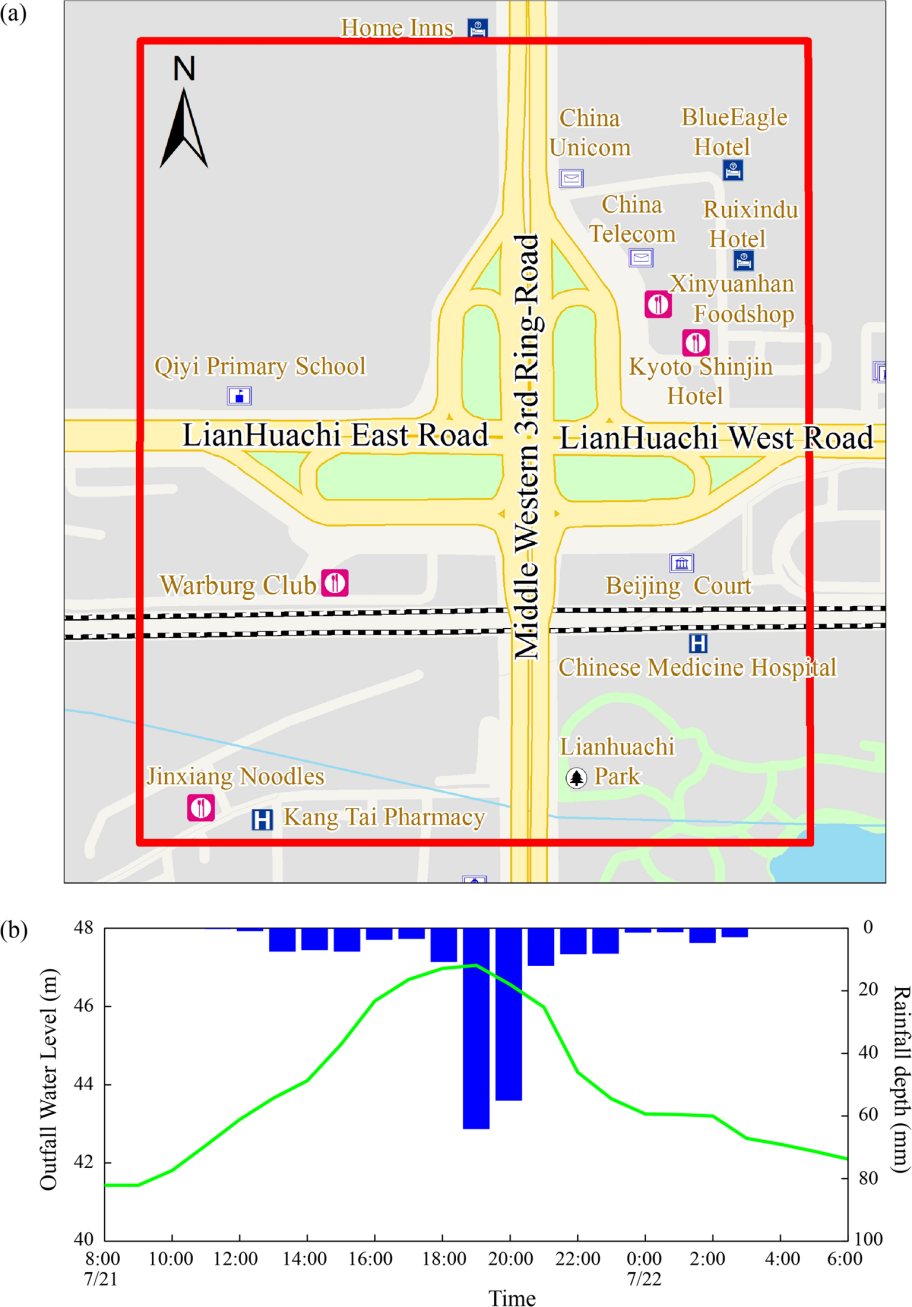
(**a**) The location of the study area is indicated by the red lines. (**b**) The outfall boundary condition for the 21st July rainfall event. The recorded water levels at *Liangshui* river are represented by the green line, and the recorded hourly rainfall depth is represented by the blue columns.

The *Lianhua* Bridge district is located in Beijing, China, which easily forms surface water. As shown in Fig. 7a, its position was at the intersection of *Lianhuachi* West Street, East Street, and Middle Western 3rd Ring Road, covering an area of 0.47 km^2^. The case study is an independent assessment based on the topography and the drainage sewer network. Different approaches were compared by using the detailed flood propagation processes as documented for July 21 rainfall event. This rainfall event occurred on July 21, 2012. As Fig. 7b shows, the total rainfall was 197 mm over 17 h, and accumulative rainfall between 18:00 and 20:00 reached a maximum of 119 mm. This was the heaviest rainstorm that Beijing had experienced in 20 years.

#### Sensitivity analysis of model parameters

The setting of model parameters can determine the accuracy of the model simulation. The main parameters, including measurability and sensitivity characteristics^37^, are summarized in Table 3.

**Table 3:** Characteristics of model parameters.

Model	Category	Parameter	Measurability (Y/N)	Sensitivity (Y/N)	Original values method
Pipe system (SWMM)	Parameters of sub-catchment	Imperv	Y	Y	Calculated by GIS based on land use type
		Slope	Y	Y	Calculated by GIS based on DEM
		Width	Y	N	Calculated by GIS
	Manning’s roughness	N-imperv	N	Y	0.012–0.022
		N-perv	N	Y	0.2–0.46
		Roughness (Pipeline)	N	Y	Determined based on pipeline materials
Surface model	Infiltration parameters	Maximum infiltration rate	Y	Y	Double-ring infiltrometer Experiments
		Stable infiltration rate	Y	Y	
		Recession coefficient	Y	Y	
	Manning’s roughness	N	N	Y	Grass:0.15–0.41; Road:0.012–0.018; Bare land: 0.05–0.17

Among the parameters, immeasurability and sensitivity mainly account for the Manning’s roughness value. The parameter sensitivity analysis is essential for the model calibration and verification process. Based on the original value range, the modified Morris screening method^38^ using Eq. (13) was adopted to carry out the sensitivity analysis. This research adopted 1-year, 5-year, 10-year, and 20-year rainfall events in Beijing to analyze the sensitivity of the total runoff and peak flow of the outfall. Analysis results are shown in Table 4. Table 4 shows that the sensitivity of parameters decreases with increases in rainfall intensity. Additionally, we found that the intensify of the rainfall event on July 21 is close to 20-year. Thus, these parameters had little impact on this rainfall event.

13 $$S = \mathop \sum \limits_{i = 1}^{N} \frac{{\left( {M_{i + 1} - M_{i} } \right)/M_{0} }}{{\left( {P_{i + 1} - P_{i} } \right)/100}}/{\text{N}}$$

where S is the factor of parameter sensitivity; ${M}_{i+1}$ and ${M}_{i}$ are the output values of the (*i* + *1*)th and *i*th run, respectively; ${M}_{0}$ is the original value of the computed value before the parameter adjustment; ${P}_{i+1}$ and ${P}_{i}$ are the percentage changes of the parameter value after the (*i* + *1*)th and *i*th run model relative to the original parameter value; N is the running time of the model. The sensitivity can be divided into four categories:$\left|S\right|\ge 1$ is the high sensitivity parameter; $0.2\le \left|S\right|<$ 1 is the sensitive parameter; $0.05\le \left|S\right|<0.2$ is the general sensitive parameter; and $0\le \left|S\right|<0.05$ is the insensitive parameter.

**Table 4:** Sensitivity analysis results of the model parameter.

Parameters	Absolute value of S				
	1-a	3-a	5-a	10-a	20-a
N-G	0.08	0.06	0.05	0.05	0.04
N-R	0.561	0.516	0.401	0.395	0.361
N-B	0.213	0.213	0.142	0.134	0.113
N-imperv	0.112	0.081	0.047	0.016	0.015
N-perv	0.107	0.078	0.045	0.013	0.012

#### Numerical simulation details

The different model linkage approaches described in “1D/2D linkage approaches” section were used to simulate the interaction flow between the two models. For the 2D overland model, the study adopted the digital elevation model (DEM) with 5 m resolution, in which a uniform regular grid system with cell size 5 × 5 m was used. The wall was taken as the surface boundary as the study area was relatively independent. In this case study, the pipe system included 178 inlets, 200 manholes, and 380 pipelines. Manning’s roughness was measured at 0.008 s m^−1/3^. A drainage pump was installed in the region with a discharge of approximately 4.1 m^3^/s to pump water into the *Liangshui* river. For the pipe system, the measured water level process was adopted for the outfall boundary condition, indicated by the green line shown in Fig. 7b. In the SF method, the land use map and pipe system were used to divide the study area to 171 sub-catchments, which were then divided based on the distribution of manholes using the Tyson polygon method. Each sub-catchment had one node that collected the surface runoff into the pipe system. The extent of flooding was simulated in order to evaluate the performance of five modeling approaches for the July 21st rainfall event. The inundation information was obtained from the Beijing Water Supplies Bureau, media articles, and photographs showing the circumstances of the July 21st rainfall event. This dataset was used to compare the accuracy of the modeled results based on five interaction methods.

#### Results and discussion

The modeled depth and extent of flooding were compared with the observed and recorded values based on five modeled methods. The robust, short-term rainfall during a two-hour period caused urban inundation in the study area because the outfall was top-lifted by the high water level. The *Liangshui* river maintained a high water level and, therefore, while the pumping stations were operating at 81.88% efficiency, they were unable to limit the surface flooding to the river.

**Figure 8:**
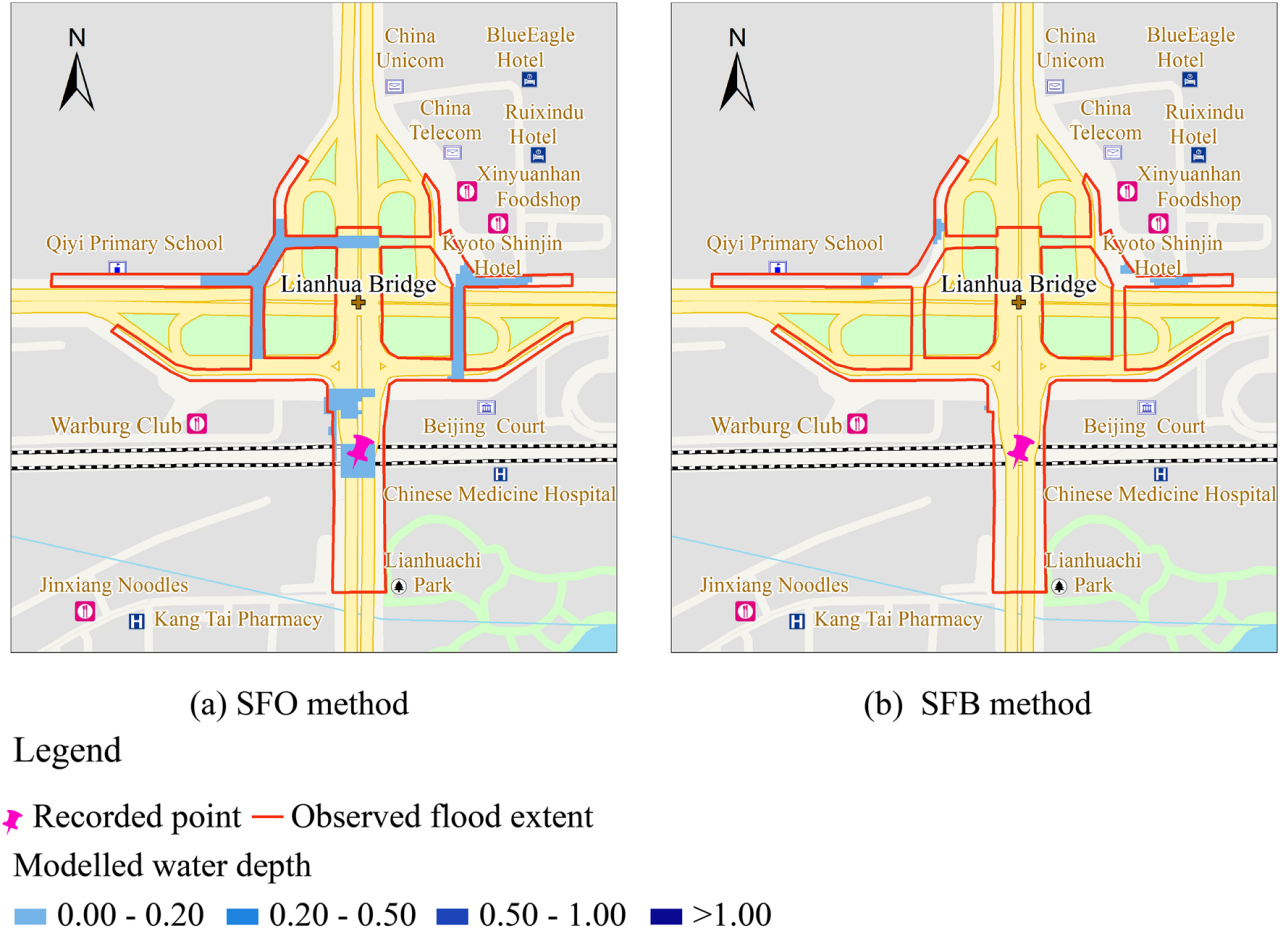
The SF method inundation areas.

Regarding the July 21st event, the observed inundation area was approximately 4.86 hectares, the recorded water depth was between 40–80 cm, and the event location was the low-lying region of the *Lianhua* Bridge district. Figure 8 provides the observed extent of flooding, indicated by the red line, and the modeled extent of flooding using the SF interaction method. The SF method assumed the rainfall applied to sub-catchments. The pipe system first collected the surface runoff, and the inundation area was induced by the overflowed water volume through the nodes. The extent of flooding areas was sporadic, as shown in Fig. 8. The results of the SF method showed a low correlation compared to the FS method, as presented in Table 5. Notably, the results of the SF simulation method failed to correctly predict the inundation area and flood depth in the case study; this was due to the 2D model neglecting the initial surface dynamics before the origin runoff entered the receiving node.

**Table 5:** Goodness of fit of five interaction methods.

Method	SFO	SFB	FSU	FSMI	FSMIC
Goodness of fit	0.239	0.027	0.891	0.917	0.915

**Figure 9:**
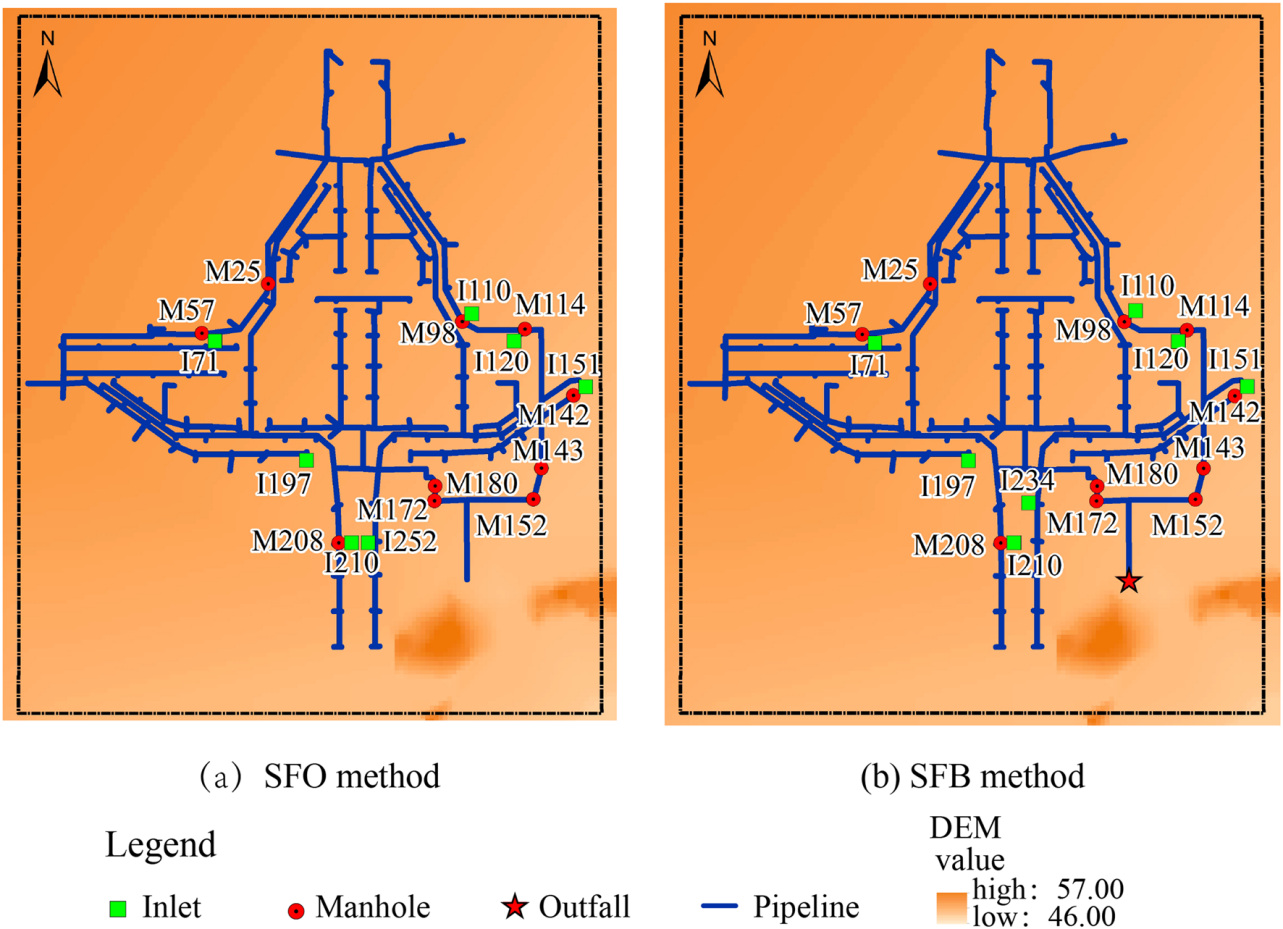
Overflow nodes of the SF method.

In the SF method, without considering the capacity of exchange nodes, the model took advantage of the runoff directly as inflow discharged into exchange nodes, where the discharge process was not elaborated. Therefore, the nodes collected an excessive discharge in the pipe system. The urban inundation areas were underestimated using the SF method. The SF method caused a higher overflow rate at the downstream pipe system, as shown in Fig. 9, because the overflow concentrated at the end of the pipe system.

**Figure 10:**
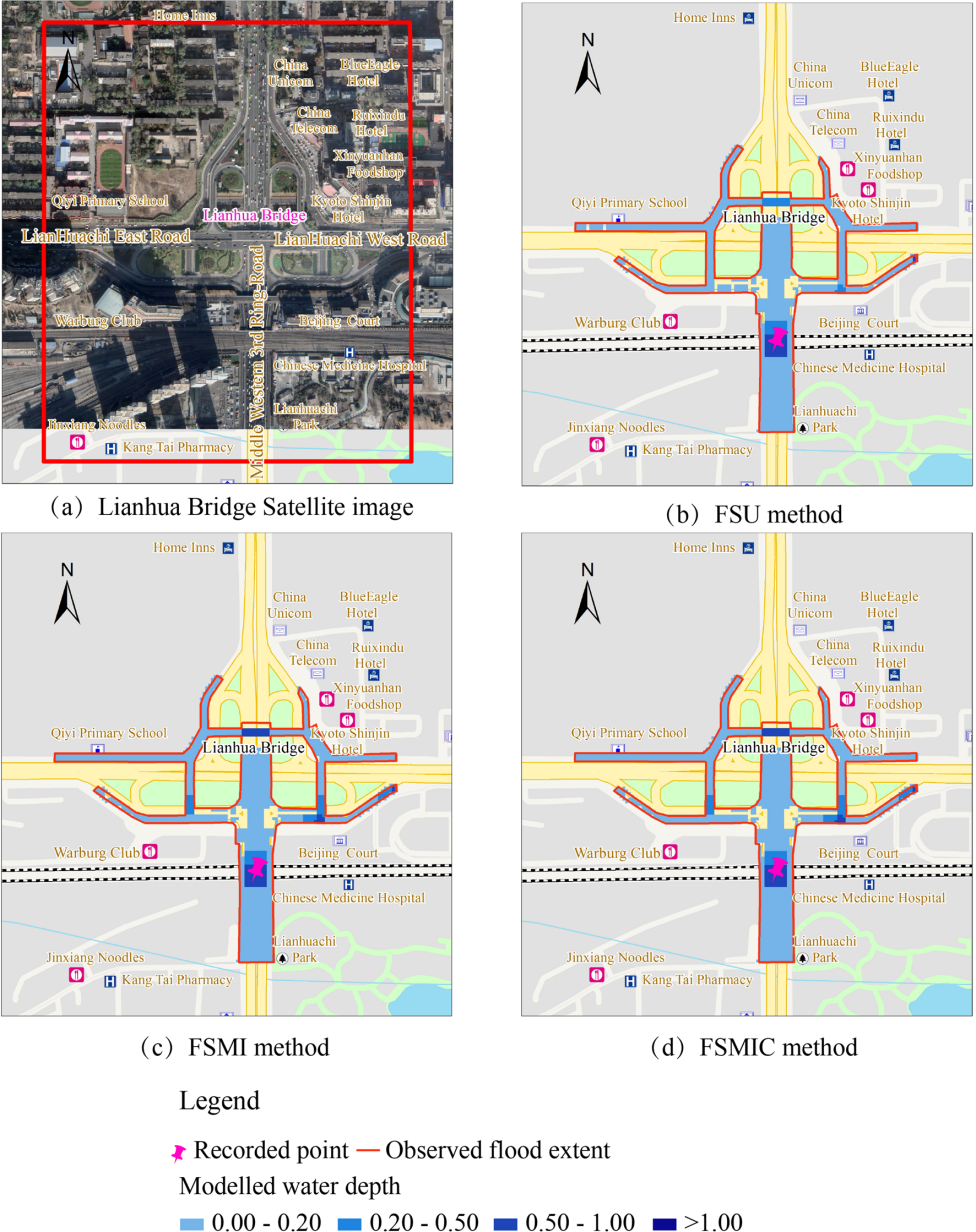
Inundation areas for the FS method.

Figure 10 shows the observed extent of the flooding indicated by the red line, and the modeled extent of the flooding using the FS interaction method. The FS method assumes the rainfall applied to surface cells. This method considered the surface flood propagation process using the hydrodynamic model, capable of simulating the initial surface runoff dynamics. As shown in Fig. 10, most inundation ranges were concentrated in the local low-lying region of the *Lianhua* Bridge district, and the modeled extent correlated with the observed flood extent based on the goodness-of-fit calculation. In this case study, the simulation results were able to predict the inundation levels.

**Figure 11:**
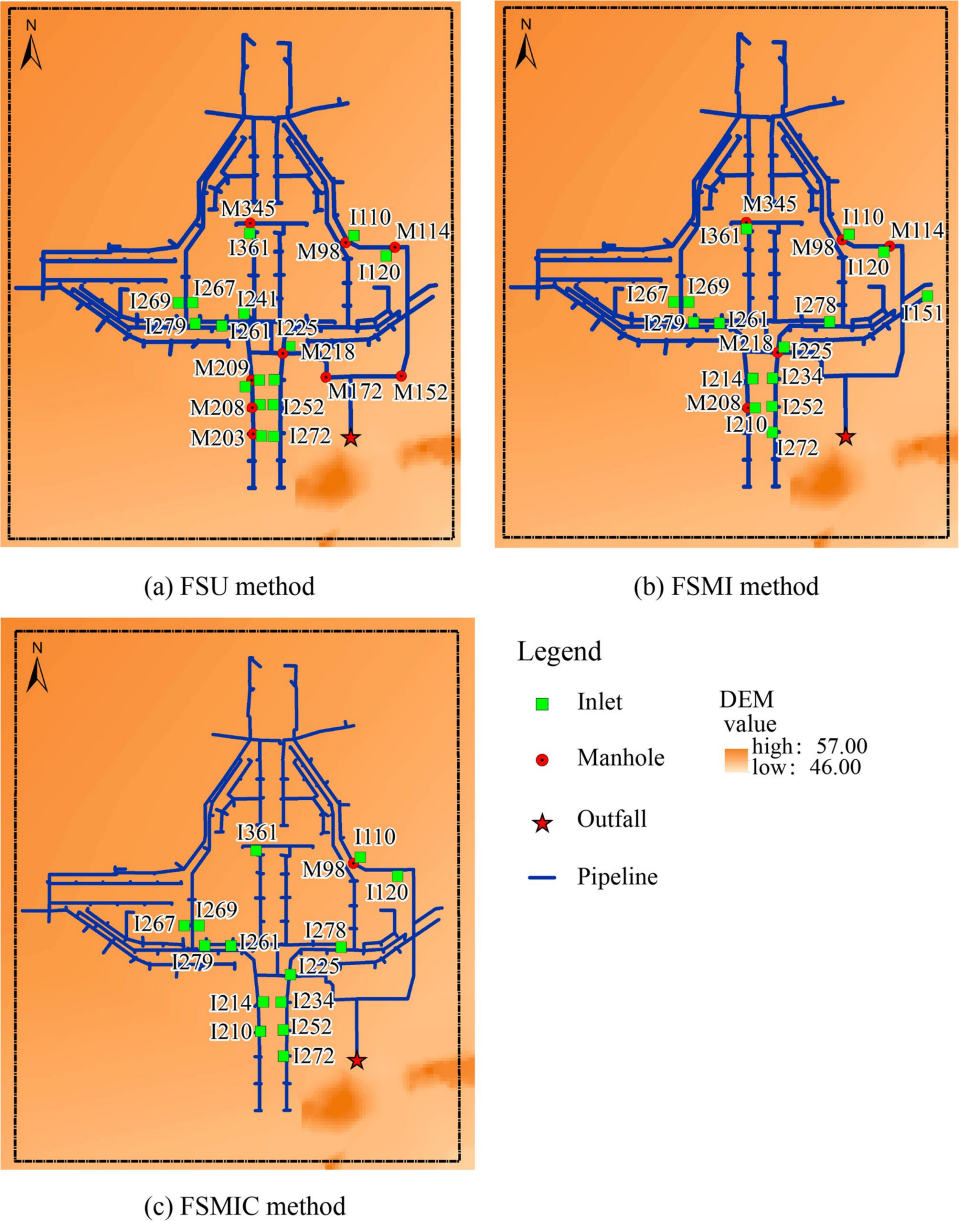
Overflowed nodes for the FS method.

**Figure 12:**
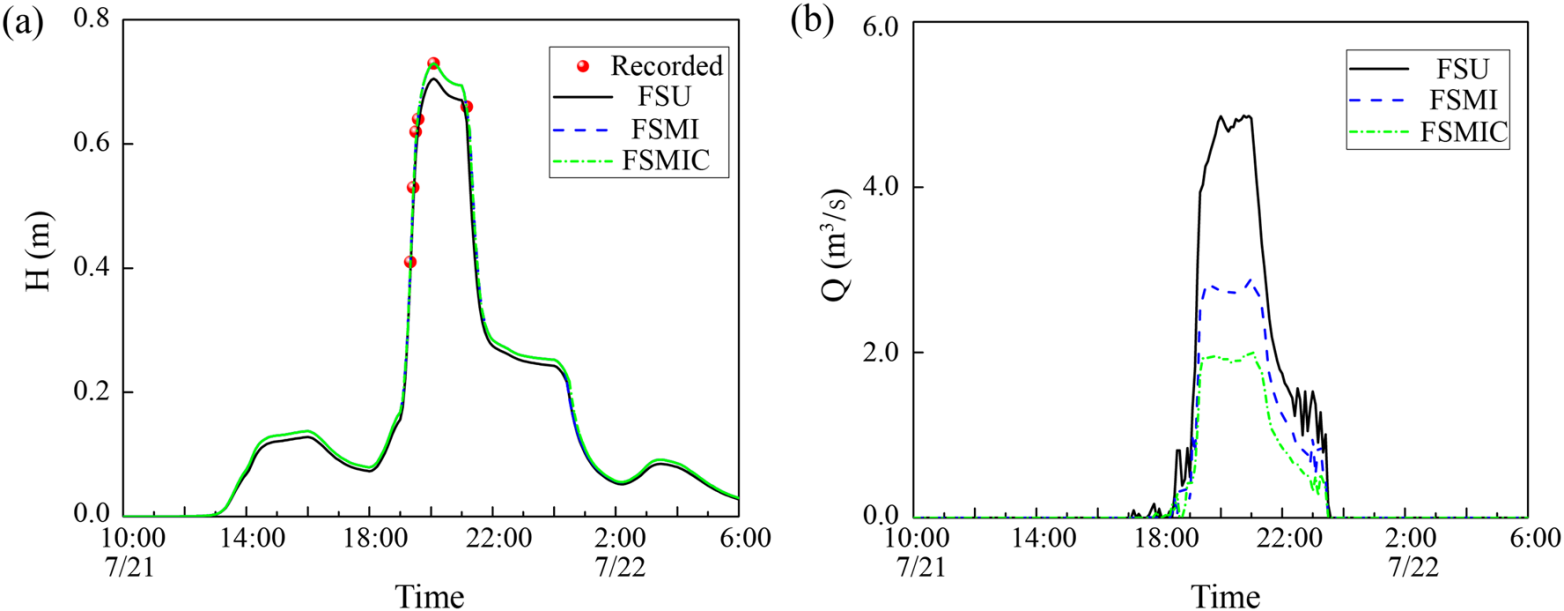
Comparison of the three FS methods. (**a**) Recorded point water depth. (**b**) M98 overflow discharge. The black solid line, the blue dash line, and the green short dash dot line respectively express the computed values of the FSU, FSMI, and FSMIC methods. The red point expresses the recorded water depth in the 721 rainfall event.

The FS method calculated the discharges from the surface to the pipe system using weir and orifice equations. The overflowed nodes were mostly concentrated in the low-lying bridge district, which correlated with the inundation area, as shown in Fig. 11. To further analyze the simulated results of these methods, the measured and modeled water depth process of the FS method is depicted in Fig. 12a, and the results of the error and correlation analysis are exhibited in Table 6, which includes the RMSE, RPE, R^2^, and NSE values, as mentioned in “Model rationality analysis method” section.

**Table 6:** Comparison of recorded and computed water depths of FS method.

Method	FSU	FSMI	FSMIC
RMSE(cm)	2.644	0.917	0.915
RPE(%)	3.456	0.085	0.056
R^2^	0.995	0.996	0.997
NSE	0.934	0.987	0.990

The FSU method simplified all exchange nodes as manholes when, in reality, the exchange nodes could have been divided into two types: inlets and manholes. Thus, the exchange nodes collected excessive discharge and delivered them to the pipe systems. This method resulted in underestimated flooding, which had higher error values and lower correlation than the other two methods. As per this table, the FMIC method had the lowest error values and highest correlation of these three methods.

Additionally, Fig. 12b depicts the overflow discharge process of these three methods. This figure shows that the overflow discharge of the FSU method is larger than that of the other two methods. Manhole covers could delay the overflow process under the overloaded condition. The FSMIC method, which was developed based on the FSMI method, was marginally better than the FSMI method because it considered manhole covers’ potential to reduce the overflowing discharge and slightly delay the overland inundation process in a manhole-overloaded region.

## Conclusion

This study found that the new urban flood model is effective and appropriate, as validated based on the experimental dataset. In this paper, the *Lianhua* Bridge case study was used to compare five interaction approaches in a real-life rainfall event. The SF method failed to correctly predict the inundation area range and flood depth, because this method neglects the capacity of exchange nodes, and initial surface runoff dynamics. In the SF method, the extent of flooding areas was quite sporadic, and this method caused a higher overflow rate at the downstream pipe system. The FS method not only calculates the discharge between the two models based on the weir and orifice equations, but also considers the initial surface runoff dynamics. The results for the FS method suggest that it may accurately predict the inundation process in the study area. However, the exchange nodes collect excessive discharge in the sewer network in the FSU model, resulting in underestimated surface flood levels and a higher overflow rate at exchange nodes in the low-lying region. Moreover, the FSU model has a larger overflow discharge than the other two methods. The FSMIC method, based on the FSMI method, is marginally better than the latter because it considers whether manhole covers reduce overflow discharge and slightly increase overland inundation in the low-lying region. In the July 21^st^ rainfall event, the rainfall intensity exceeded the design standard of the pipe network, which has only happened once in ten years. The pipe network then overloaded and overflowed. In conclusion, the FSMIC is the most predictive interaction method.
